# Prognostic superiority of volumetric PET parameters (MTV and TLG) over SUV in advanced non-small-cell lung cancer: a systematic review and meta-analysis of univariate and multivariate evidence

**DOI:** 10.3389/fimmu.2026.1896967

**Published:** 2026-08-25

**Authors:** Jinwen Han, Hanxiang Zhang, Xianhong He, Zhimin Wang

**Affiliations:** 1 The first Clinical Medical College of Gansu University of Chinese Medicine (Gansu Provincial Hospital), Lanzhou, Gansu, China; 2 PET/CT Center, Gansu Provincial Hospital, Lanzhou, Gansu, China

**Keywords:** [18F] FDG PET/CT metabolic parameters, immune checkpoint inhibitors, immunotherapy, non-small-cell lung cancer, prognosis

## Abstract

**Purpose:**

To systematically assess the prognostic significance of baseline ^18^F-fluorodeoxyglucose positron emission tomography/computed tomography ([^18^F] FDG PET/CT) metabolic parameters in patients with non-small cell lung cancer (NSCLC) undergoing immune checkpoint inhibitor (ICI) therapy.

**Methods:**

A systematic search of five core databases was conducted up to March 2026. Relevant data concerning NSCLC patient characteristics, metabolic [^18^F] FDG PET/CT parameters, and survival outcomes were extracted. Hazard ratios (HRs) were pooled to assess the prognostic significance of maximum standardised uptake value (SUVmax), mean standardised uptake value (SUVmean), metabolic tumour volume (MTV), and total lesion glycolysis (TLG) regarding overall survival (OS) and progression-free survival (PFS).

**Results:**

A total of 33 studies were eligible for inclusion. Univariable analysis showed no prognostic value for SUVmax and SUVmean. Conversely, elevated MTV and TLG were associated with inferior PFS (MTV: HR = 1.71,95% CI: 1.39–2.11; TLG: HR = 1.84, 95%CI: 1.31-2.57) and OS (MTV: HR = 1.80, 95%CI: 1.40-2.33; TLG: HR = 1.92, 95%CI: 1.18-3.12) (*P* < 0.01), particularly when cut-off values ranged of 50-100cm^3^ for MTV (PFS: HR = 1.71, 95%CI: 1.16-2.52; OS: HR = 2.23, 95%CI: 1.42-3.51). Multivariable Cox regression analysis further identified MTV as a robust independent predictor of both PFS (HR = 1.21, 95%CI: 1.07-1.36) and OS (HR = 1.14, 95%CI: 1.03-1.26). While TLG maintained independent prognostic significance for PFS (HR = 1.46, 95%CI: 1.10-1.94), it failed to reach statistical significance regarding OS (HR = 1.00, 95%CI: 0.99-1.01) (*P* > 0.01). Notably, subgroup analyses demonstrated that the prognostic efficacy of MTV and TLG was further pronounced in patients presenting with Stage IV disease, those undergoing first-line treatment, or those managed with chemoimmunotherapy regimens.

**Conclusion:**

Baseline MTV is an independent prognostic factor for PFS and OS in NSCLC patients treated with ICIs, with a cut-off value within the range of 50–100 cm³ demonstrating optimal predictive performance. In contrast, the predictive significance of TLG for OS was not maintained after multivariable adjustment.

**Systematic review registration:**

https://www.crd.york.ac.uk/PROSPERO/view/CRD420261375745, identifier CRD420261375745.

## Graphical Abstract

**Figure:**
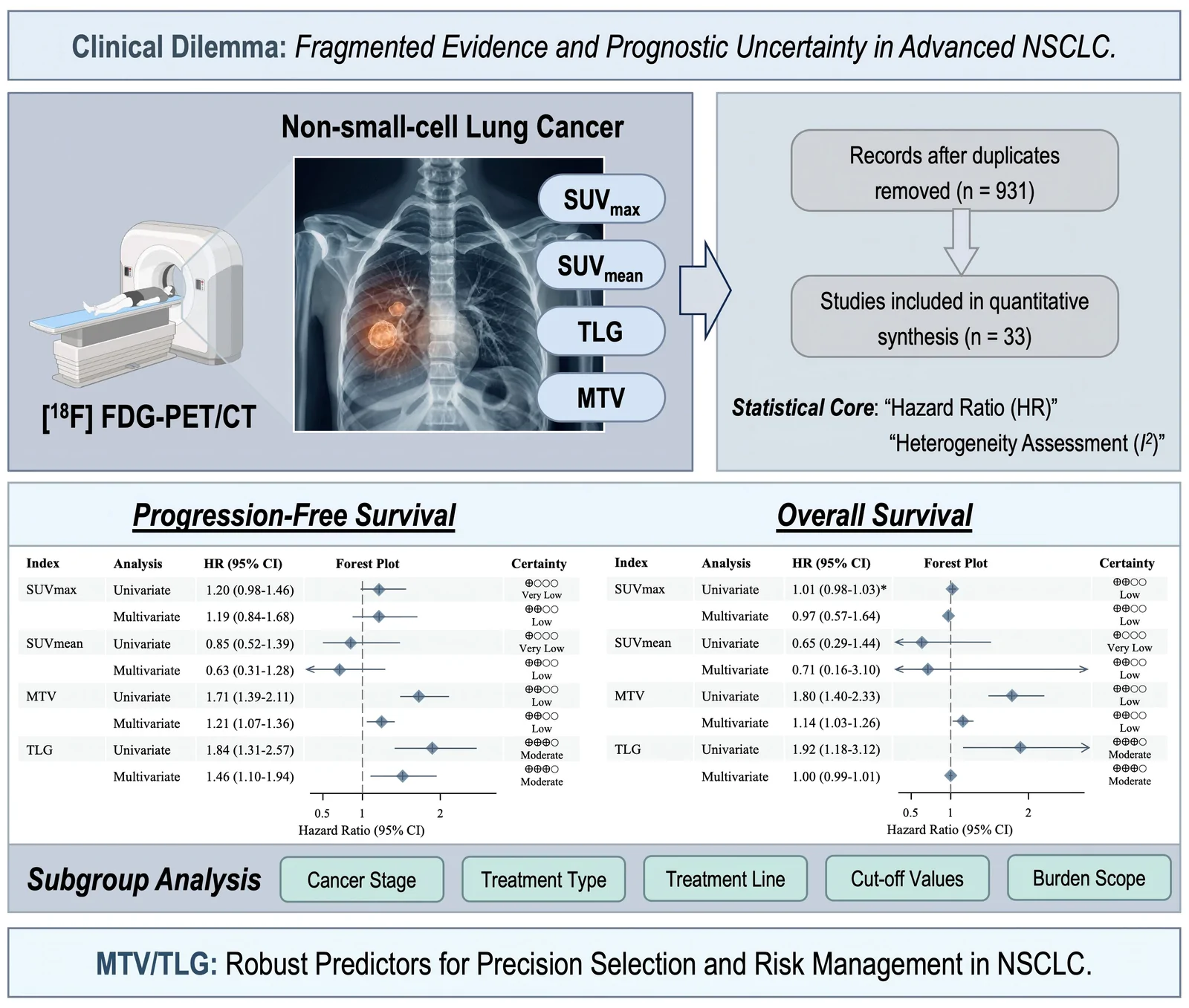
Prognostic superiority of volumetric PET parameters (MTV and TLG) over SUV in advanced non-small-cell lung cancer.

## 1 Introduction

Lung cancer remains a major global health burden, causing approximately 2 million new cases and 1.76 million deaths annually (1). Histologically, it is classified as small-cell lung cancer or non-small-cell lung cancer (NSCLC), with NSCLC accounting for approximately 85% of cases (2). Despite standard treatments, including surgery and chemoradiotherapy, the management of early-stage NSCLC remains challenging because of occult nodal involvement, heterogeneous radiological features, and specific molecular alterations, which may compromise treatment outcomes and contribute to poor prognosis (3, 4).

Immune checkpoint inhibitors (ICIs), particularly programmed cell death protein 1/programmed death-ligand 1 (PD-1/PD-L1) inhibitors, have become a first-line treatment for advanced NSCLC. Used alone or with chemotherapy, ICIs significantly improve progression-free survival and overall survival (5, 6). However, their clinical application remains limited by difficulties in identifying responders, evaluating treatment response, and managing immune-related adverse events. PD-L1 expression has restricted predictive accuracy because of spatiotemporal heterogeneity and non-standardized assays, which inadequately capture the dynamic tumor immune microenvironment (7). Moreover, the morphology-based Response Evaluation Criteria in Solid Tumors version 1.1 (RECIST 1.1) cannot reliably distinguish pseudoprogression from true progression (8). Robust biomarkers capable of predicting treatment response and clinical outcomes are therefore needed to identify patients most likely to benefit from ICIs and guide personalized treatment strategies.

With the increasing use of immune checkpoint inhibitors (ICIs), ^18^F-fluorodeoxyglucose positron emission tomography/computed tomography ([^18^F] FDG PET/CT) has become valuable for accurate staging and prognostic assessment in NSCLC (9, 10). By detecting increased glucose transporter expression and glycolytic activity in tumor cells, this modality enables metabolic tumor burden to be characterized using standardized uptake values (SUVs), metabolic tumor volume (MTV), and total lesion glycolysis (TLG). However, their prognostic value remains controversial. Some studies have identified MTV and TLG as stronger independent prognostic factors than SUV (11–14), whereas others found that only TLG or SUV_max remained independently prognostic after confounder adjustment (15). Clinical implementation is further limited by non-standardized cutoff values and insufficient evidence across treatment lines and regimens. This systematic review and meta-analysis therefore aimed to clarify the prognostic value of baseline [^18^F] FDG PET/CT parameters and inform patient selection for immunotherapy in NSCLC.

## 2 Materials and methods

This study was conducted in accordance with the PRISMA (Preferred Reporting Items for Systematic Reviews and Meta-Analyses) statement (16). The study protocol was duly registered with the PROSPERO (CRD420261375745).

### 2.1 Literature search

A comprehensive search was conducted by two senior nuclear medicine investigators, who independently searched the PubMed, Web of Science, Embase, Cochrane Library, and Chinese National Knowledge Infrastructure (CNKI) databases from inception to March 2026. The search encompassed full-text studies involving human subjects published in English and Chinese. The search strategy employed a combination of key terms, including “non-small cell lung cancer”, “PET/CT”, and “immune checkpoint inhibitors”. The search strategy for PubMed is provided in the **Supplementary Table 1** as a representative example. Following an initial screening of titles and abstracts, the eligibility of candidate articles was assessed via full-text review. Any discrepancies were resolved through arbitration by a third senior nuclear medicine expert.

### 2.2 Inclusion and exclusion criteria

The inclusion criteria were structured according to the PICOS framework:

Patients (P): Histologically or pathologically confirmed NSCLC. Intervention (I): Baseline [^18^F] FDG PET/CT scan prior to the initiation of ICI therapy (monotherapy or multimodal regimens involving chemotherapy or radiotherapy). Comparator (C): No specific control group; the study focused on prognostic stratification based on baseline metabolic indices. Outcomes (O): overall survival (OS) or progression-free survival (PFS) reported as primary or secondary endpoints. Study Design (S): Prospective or retrospective clinical studies.

Exclusion criteria: Overlapping cohorts, missing baseline data, or insufficient follow-up and survival outcomes.

### 2.3 Methodology and quality assessment of evidence

Two investigators independently appraised the risk of bias using the Newcastle–Ottawa Scale (NOS) (17). Each study was subjected to critical appraisal across three core dimensions: cohort selection, inter-group comparability, and outcome measurement. The results were cross-referenced between the reviewers, with any discrepancies resolved through consensus or, where necessary, via arbitration by a third investigator to ensure the objectivity of the assessment.

The certainty of the evidence for each outcome was evaluated using the GRADE (Grading of Recommendations, Assessment, Development and Evaluations) framework (18, 19). Given the observational design of all included studies, the baseline evidence quality was initially categorised as “low”. Outcomes were prioritised as either “critical” or “important” to clinical decision-making.

The evaluation was subject to downgrading across five domains: risk of bias (deemed non-serious following multivariable adjustment); inconsistency (characterised by significant statistical heterogeneity, *I²* > 50%); indirectness; imprecision and publication bias (evaluated via visual inspection of funnel plot asymmetry alongside Begg’s and Egger’s tests, *P* < 0.01). Conversely, the potential for upgrading was assessed based on a large magnitude of effect, the presence of a dose-response gradient, and the anticipated influence of residual confounding.

Two investigators independently performed these assessments, resolving any discrepancies via consensus. Ultimately, the overall certainty of evidence was classified as “high”, “moderate”, “low”, or “very low”.

### 2.4 Data extraction

Data extraction was performed independently by two investigators, encompassing the following parameters: 1) Study metadata: First author, publication year, and country of origin. 2) Baseline characteristics and treatment regimens. 3) Optimal thresholds for MTV and TLG: Cut-off values determined via log-rank statistics or ROC curve analysis were prioritised; failing these, median splits were employed. 4) Prognostic outcomes: OS and PFS, specifically extracting univariate or multivariate hazard ratios (HRs) and their associated 95% confidence intervals (CIs), whether reported directly or calculated through indirect estimation. Any discrepancies between the primary reviewers were resolved through consensus or by consultation with a third senior nuclear medicine specialist.

### 2.5 Statistical analysis

Cumulative survival outcomes were evaluated using HRs and their corresponding 95% CIs as the primary effect size measures. Both univariate and multivariate HRs were pooled for SUVmax, SUVmean, MTV, and TLG to assess their independent prognostic value.

The overall effect size was calculated using either a fixed-effects or random-effects model, depending on the presence of statistical heterogeneity. Heterogeneity across studies was quantified using the *Cochran Q test* and the *I^2^* statistic. In cases of significant heterogeneity (*I^2^*> 50% or *P* < 0.05 for the *Q test*), the random-effects model (DerSimonian-Laird method) was prioritized to provide a more conservative estimate; otherwise, the fixed-effects model (Mantel-Haenszel method) was applied.

To explore the potential drivers of heterogeneity, subgroup analyses were performed for parameters reported in at least five studies, categorized by cancer stage, treatment type, and treatment line. For indicators supported by more than ten studies, sensitivity analyses were conducted using the leave-one-out approach to examine the robustness of the pooled results and identify any single study exerting a disproportionate influence. Publication bias was rigorously assessed using Begg’s and Egger’s tests, complemented by visual inspection of funnel plots.

All statistical procedures were performed using R software (version 4.5.3). All tests were two-sided, and a P-value < 0.05 was defined as the threshold for statistical significance.

## 3 Results

### 3.1 Study selection and research characteristics

**Figure 1:**
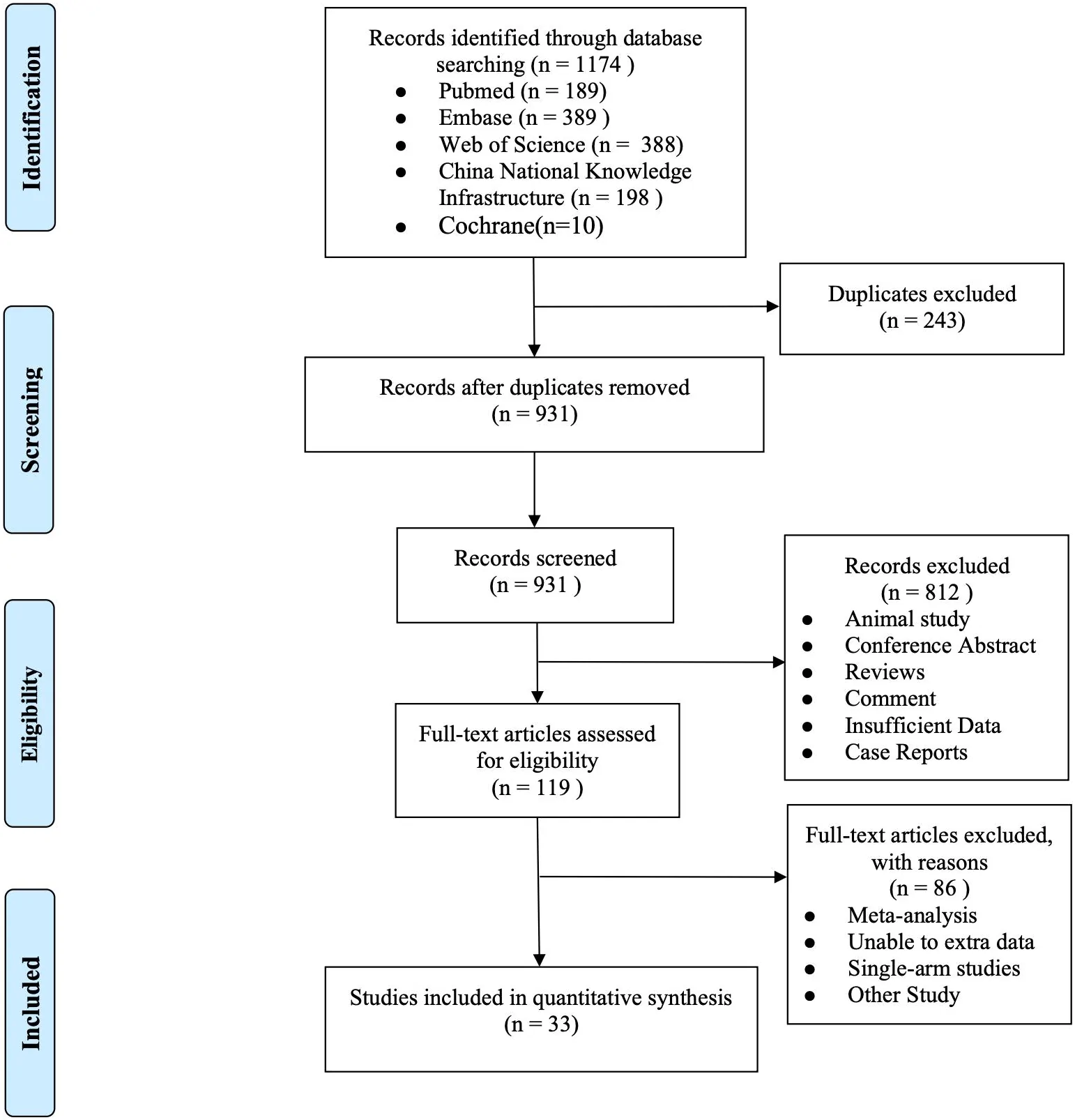
Flow chart of selection studies and specific reasons for exclusion.

**Figure 1** delineates the literature screening process. Ultimately, 33 of the 1174 identified records met the inclusion criteria (**Table 1**). The aggregated cohort predominantly comprised patients with advanced disease (Stage IIIB–IV) receiving ICI therapy. The specific treatments involved monotherapy (n=22), chemoimmunotherapy (n=3), and unspecified regimens (n=8). These therapies were administered in first-line (n=15), mixed (n=13), or undefined (n=5) settings. For lesion delineation, all studies utilized independent semi-automatic segmentation with manual corrections to exclude physiological or inflammatory FDG uptake. Regarding study quality, although overall tracking was adequate, 24% of the studies reported insufficient follow-up duration and 18% suffered from compromised follow-up integrity.

**Table 1:** The principal characteristics and details of eligible articles.

Author	Sample size	Year	Country	Study design	Age	Stage	Treatment	Treatment Line	Cut-off Values	Burden scope	Detection time	Response assessment criteria	Parameters	Endpoint	Follow-up (months)
Kaira	24	2017	Japan	Prospective study	66 (47-82)	III-IV	ICI	Later-line	Unreported	Whole-body	Baseline+post-treatment	RECIST1.1/PERCIST	SUVmax/MTV/TLG	OS PFS	10.5 (3.6-17)
Seban 1	80	2019	France	Retrospective study	61.9 (34.2-84.8)	IIIB-IV	ICI	Later-line	MTV: 75	Whole-body	Baseline	RECISTv1.1	SUVmax/TMTV/TLG/NLR/LDH levels	OS PFS	11.6 (7.7-15.5)
Takada	89	2019	Japan	Retrospective study	67 (36-88)	IIIB-IV	ICI	Total	Unreported	Whole-body	Baseline	RECISTv1.1	SUVmax	OS PFS	7.5 (0.2-31.0)
Valentinuzzi	30	2020	Slovenia	Prospective study	65 (46-77)	IV	ICI	Total	Unreported	Whole-body	Pre/post-treatment	iRECIST/iRADIOMICS/PD-L1TPS	SUVmax/Volumn/Sum Entropy	OS	21.4
Oded Icht	58	2020	Israel	Retrospective study	65 (59-71)	IIIB-IV	ICI	Total	MTV: 12.95	Whole-body	Baseline	RECIST1.1	SOML/MTV	OS PFS	Unreported
Castello 1	33	2020	Italy	Prospective study	77 (51-86)	IV	ICI	Total	MTV: 68 TLG: 392	Whole-body	Baseline+post-treatment	RECIST1.1/EORTC	SUVmax/SUVmean/MTV/TLG/CTC	OS PFS	13.2 (4.9-21.6)
Park	24	2020	South Korea	Retrospective study	58 (29-88)	IIIa/IIIb/IV	ICI	Total	Unreported	Single-hottest	Baseline+post-treatment	PERCIST1.0/EORTC/RECIST1.1	SUVpeak/SUVmax/MTV/TLG	PFS	4.2 (0.93-15.87)
Seban 2	63	2020	France	Retrospective study	65 (37-86)	IIIB-IV	ICI	First line	MTV: 84 TLG: 597.2	Whole-body	Baseline	RECISTv1.1	SUVmean/SUVmax/Tmtv/TLG	LTB OS PFS	13.4 (1.5-29.1)
Chardin	75	2020	France	Retrospective study	64 (42-83)	III-IV	ICI	Total	MTV: 36.5 TLG: 267	Whole-body	Baseline	RERCIST	SUVmax/SUVpeak/MTV/TLG	OS ETD	12.3 (6.1-19.3)
Hashimoto	85	2020	France	Retrospective study	70 (38-86)	IV	ICI	Later-line	MTV: 5* TLG: 20*	Whole-body	Baseline	RECISTv1.1	SUVmax/SUVmean/MTV/TLG	OS PFS	Unreported
Yamaguchi	48	2020	Japan	Prospective study	69 (47-86)	IV	ICI	First line	MTV: 268* TLG: 697*	Whole-body	Baseline	RECISTv1.1	SUVmax//MTV/TLG	OS PFS	11.5 (1-29.5)
Andraos	124	2020	UK	Retrospective study	67 (38-88)	IV	Undefined	First line	MTV: 88*	Whole-body	Baseline	RECIST1.1	MTV	OS PFS	17
Seban 3	63	2020	France	Retrospective study	65 (37-86)	IIIB-IV	ICI	First line	MTV: 75	Whole-body	Baseline	RECIST1.1	TMTV/SUVmax/SUVmean	OS PFS	13.4 (9.0-17.9)
Castello 2	50	2021	Italy	Prospective study	73	IV	ICI	Total	MTV: 63.7 TLG: 330.1	Whole-body	Baseline	iRECIST/RECIST	MTV/dNLR/SUVmax/SUVmean/TLG	OS	12.4 (8.7-15.2)
Bauckneht	45	2021	Italy	Retrospective study	70.6 (50.3-81.5)	IIIB-IV	ICI	Later-line	Unreported	Whole-body	Baseline	RECIST1.1/irRC/iRECIST/PERCIST	SUVmax/MTV/TLG/SII/NLR/dNLR/LMR	OS	9.43
Lang	85	2021	Austria	Retrospective study	64 (38-81)	IV	Undefined	First line	Unreported	Whole-body	Baseline	RECISTv1.1	SUVmax/SUVmean/MTV/TLG/BLR	OS PFS DCR	12
Vekens	30	2021	Belgium	Retrospective study	67 (41-92)	IV	ICI	First line	MTV: 123.9 TLG: 802.6	Whole-body	Baseline+post-treatment	RECISTv1.1	SUVmax/SUVpeak/TMTV/TLG/SUVmean	OS PFS ORR	20 (4.2-37.6)
Monaco	92	2021	Italy	Retrospective study	70 (62-75)	IA-IV	ICI	Total	MTV: 94.9 TLG: 542.6	Whole-body	Baseline	RECIST1.1	SUVmax/SUVmean/wMTV/wTLG	OS PFS	Unreported
Eude	65	2022	France	Retrospective study	62.7 (40.2-90.5)	III-IV	ICI	First line	MTV: 133*	Whole-body	Baseline	RECIST1.1	MTV/NTV/TTV	OS	12
Kim	52	2022	Korea	Retrospective study	63 (33-84)	III-IV	ICI+Chemotherapy	First line	Unreported	Whole-body	Baseline	RECIST1.1	SUVmax/SUVmean/MTV/TLG	OS PFS	16.7
Kudura	50	2022	Germany	Retrospective study	67.5 (30-90)	I-IV	ICI	Total	MTV: 13.5 TLG: 71.4	Primary	Baseline	RECIST1.1	SUVmax/SUVmean/MTV/TLG	OS PFS CB	18.93
Silva	98	2022	Brazil	Retrospective study	67.5 (30-90)	III-IV	Undefined	Total	Unreported	Whole-body	Baseline	RECIST1.1	SUVmax//wMTV/Wtlg	OS PFS	Unreported
Kosuke Hashimoto	45	2022	Japan	Retrospective study	67 (37-77)	IV	ICI+Chemotherapy	First line	MTV: 65.3* TLG: 500.3*	Whole-body	Baseline	RECIST1.1	SUVmax/SUVmean/MTV/TLG	OS PFS	13.9 (1.4-31.6)
Rizzo	43	2023	Italy	Retrospective study	67 (61-72)	IV	ICI	First line	MTV: 34.62* TLG: 207.5*	Whole-body	Baseline	RECISTv1.1	MTV/TLG	OS PFS	18.2
Grambozov	48	2023	Austria	Retrospective study	67 (57-83)	I-IV	Undefined	Later-line	MTV: 22 TLG: 107	Primary	Baseline	RECIST1.1	SULpeak/SUVmax/MTV/TLG	OS PFS	16.7 (0.6-68)
Feng Yawen	34	2023	China	Retrospective study	62.5	III-IV	ICI	First line	MTV: 112.3* TLG: 391.9*	Whole-body	Baseline	RECIST1.1	TLG/MTV/SUVmax	OS PFS	16.47 (3.36-24.97)
Kong Dexian	105	2023	China	Retrospective study	61	IIIB-IV	ICI+Chemotherapy	First line	MTV: 22.54* TLG: 102*	Primary	Baseline	RECIST	SUVmax/MTV/TLG/SII/NLR/dNLR/LMR	OS PFS	17.5 (1-63)
Daria Kifjak	87	2024	Austria	Prospective study	62 ± 10	III-IV	Undefined	Total	MTV: 112 TLG: 690	Whole-body	Baseline	RECIST/iRECIST/PERCIST	SUVmaxMTV/TLG	OS PFS	11.4
M. Peng	143	2025	China	Retrospective study	62 (56-68)	III-IV	Undefined	First line	MTV: 15 TLG: 117.4	Primary	Baseline	iRECIST	SUVmax/SUVmean/MTV/TLG/WBC/PCT	OS PFS	Unreported
Xiuli Tao	35	2025	China	Retrospective study	62 (48-70)	IA-IIIB	Undefined	First line	Unreported	Primary	Baseline + post-neoadjuvant	iRECIST	SULmax/SULpeak/MTV/TLG/SULmax	OS PFS	62.6
Gao Yi	158	2025	China	Retrospective study	61.36 ± 6.78	IIIB-IV	ICI	Total	MTV: 120.2* TLG: 1498.3*	Whole-body	Baseline	RECIST1.1	SUVmax/MTV/TLG/miR-345/miR-20a	OS PFS	18 (10-24)
Luo	115	2025	China	Retrospective study	65 (28-79)	III-IV	Undefined	First line	MTV: 120.3* TLG: 434.2*	Primary	Baseline	RECIST1.1	SUVmax/MTV/TLG/MTVwb/TLGwb	OS PFS	25
Kolkiran	187	2025	Turkey	Retrospective study	66 (60-70)	IV	ICI	Total	Unreported	Whole-body	baseline	RECIST1.1	SUVmax/SUVmax-IPI	OS PFS	28

*The optimal cut-off value was primarily determined using Receiver Operating Characteristic (ROC) curve analysis or the log-rank test; Total:including first or later line treatment, and second or later line treatment.

Metabolic Parameters and Threshold Optimization: To evaluate the prognostic impact on PFS and OS, each study stratified patients into high- and low-risk groups based on MTV or TLG. The determination of optimal cut-off values varied across the literature: the median value was the most frequent threshold (n=17), followed by Receiver Operating Characteristic (ROC) curve analysis (n=10) and the log-rank test (n=2). Methodological details were unavailable for the remaining four studies.

### 3.2 Quality assessment and GRADE assessment

**Figure 2:**
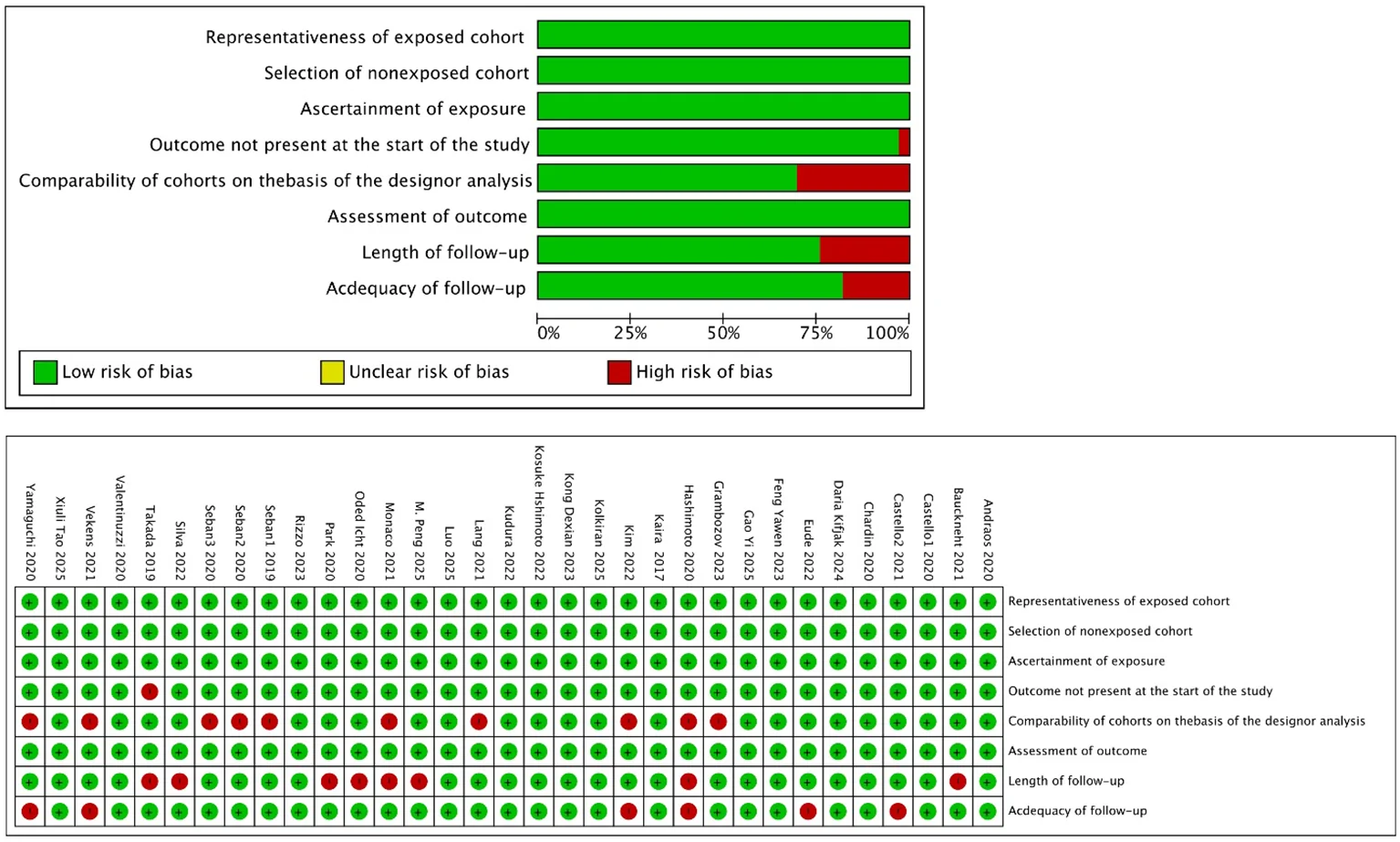
Risk of bias assessment of included cohort studies using the Newcastle-Ottawa scale (NOS).

The majority of the included studies exhibited robust methodological quality, particularly concerning cohort representativeness, exposure ascertainment, and outcome assessment. Nevertheless, specific limitations were noted: inadequate follow-up duration hampered eight studies (24%), while six (18%) suffered from suboptimal follow-up integrity, and one (3%) failed to effectively exclude baseline events. A comprehensive summary of the risk-of-bias assessment is delineated in **Figure 2**.

Following GRADE criteria, the certainty of evidence was evaluated for eight outcomes, including OS and PFS, respectively. While indirectness was minimal, the evidence was downgraded to moderate to very low certainty due to significant risk of bias, inconsistency, and imprecision (**Supplementary Table 2**).

### 3.3 Progression-free survival

#### 3.3.1 SUVmax/SUVmean

**Figure 3:**
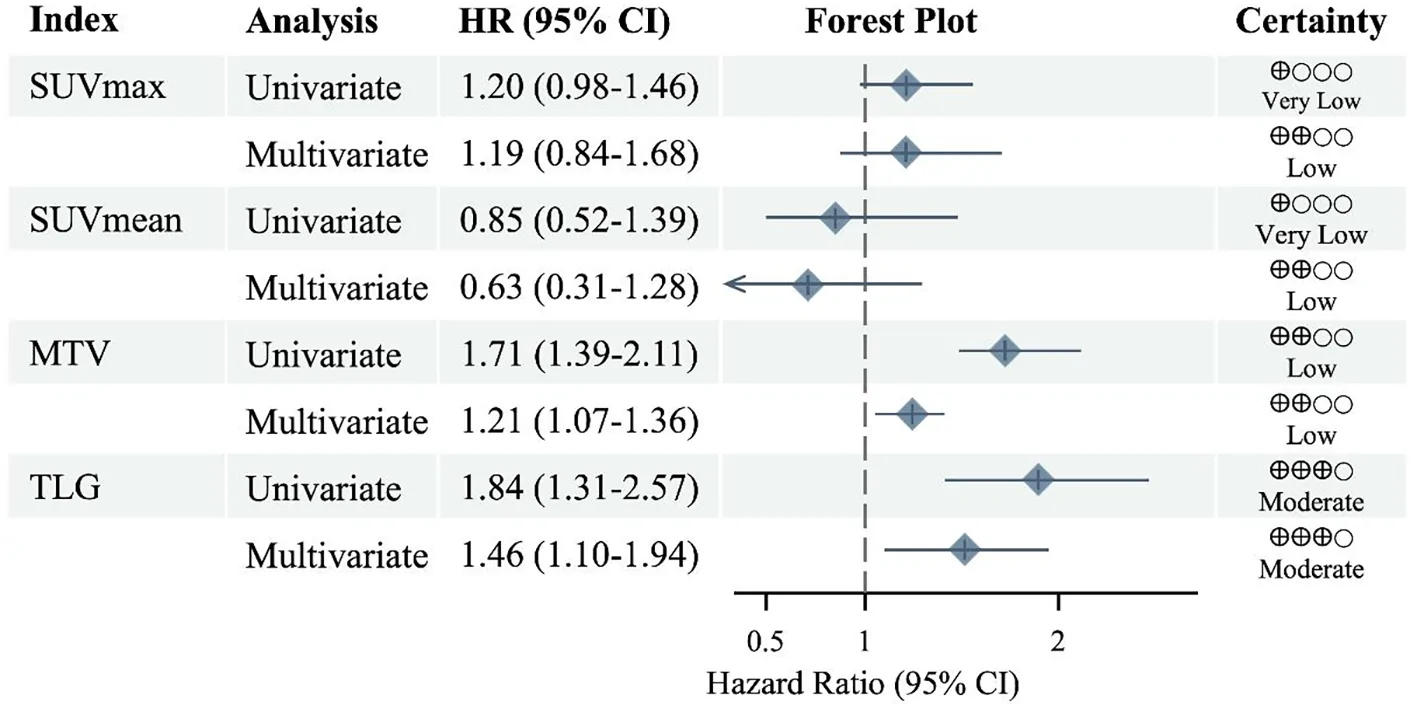
Forest plot detailing the association between PET/CT metabolic parameters and progression-free survival (PFS).

Based on the outcome measures for SUVmax and SUVmean, the univariate analysis encompassed 13 studies (n=1,073) (20–32) and 6 studies (n=416) (22, 24, 25, 32–34), respectively (**Figure 3**). Neither SUVmax nor SUVmean were significant associated with PFS (**Supplementary Figures 1A**, **2A**). For the SUVmax, sensitivity analysis via a random-effects model confirmed the robustness of the pooled estimates (**Supplementary Figure 1C**). Additionally, Begg’s and Egger’s tests yielded no evidence of significant publication bias (**Supplementary Figure 1D**). These findings were further corroborated by subsequent multivariate analysis, which remained highly consistent with the univariate results (**Figure 3**; **Supplementary Figures 1B**, **2B**).

#### 3.3.2 MTV/TLG

A total of 17 studies (n = 1,273) (20, 22–27, 30–33, 35–41) and 9 studies (n = 828) (22–27, 29, 31, 32, 38, 39) were included for the assessment of MTV and TLG, respectively (**Figure 3**). Both high parameters were significantly associated with inferior PFS (**Supplementary Figures 3A**, **4A**). TLG yielded a pooled HR of 1.84 (95% CI 1.31–2.57; GRADE: moderate), while MTV yielded an HR of 1.71 (95% CI 1.39–2.11; GRADE: low). Despite evidence of publication bias from Egger’s test (**Supplementary Figures 3D**, **4D**), leave-one-out sensitivity analysis showed consistent statistical significance (P < 0.001), further validating the reliability of these results (**Supplementary Figures 3C**, **4C**).

For the multivariable Cox regression analysis, 14 studies (n = 967) (23, 24, 28, 29, 31, 33, 34, 37, 39, 42–46) for MTV and 9 studies (n = 579) (28, 29, 31, 42, 44–48) for TLG were evaluated (**Figure 3**). MTV (HR 1.21, 95% CI 1.07–1.36; GRADE: low) and TLG (HR 1.71, 95% CI 1.10–1.94; GRADE: moderate) remained significant predictors of PFS (**Supplementary Figures 3B**, **4B**). The pooled HR for TLG was consistently higher than for MTV. Random-effects modelling and Begg’s and Egger’s tests showed that multivariable results for MTV were consistent with univariate estimates (**Supplementary Figures 3E, F**).

### 3.4 Overall survival

#### 3.4.1 SUVmax/SUVmean

**Figure 4:**
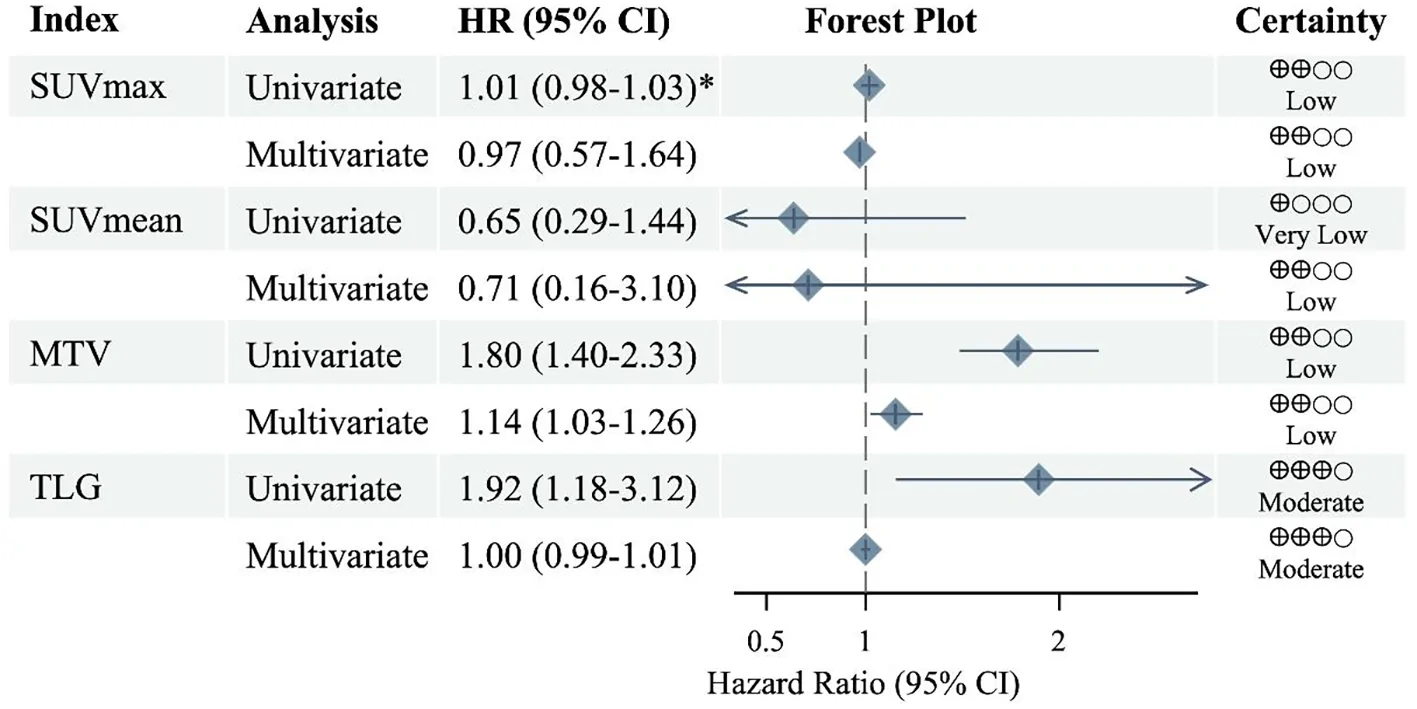
Forest plot detailing the association between PET/CT metabolic parameters and overall survival (OS).

For the univariate analysis, 15 studies (n=1,263) (20–22, 24, 25, 27, 29, 32, 33, 39–41, 49–51) for SUVmax and 6 studies (n=496) (22, 24, 25, 32, 34, 40) for SUVmean were evaluated (**Figure 4**). Neither parameter showed a significant association with OS (**Supplementary Figures 5A**, **6A**). SUVmax demonstrated lower heterogeneity compared to SUVmean. Its pooled HR remained stable during leave-one-out sensitivity analysis (**Supplementary Figure 5C**), with no evidence of publication bias (**Supplementary Figure 5D**). These non-significant findings persisted in the subsequent multivariable analysis (**Figure 4**; **Supplementary Figures 5B**, **6B**).

#### 3.4.2 MTV/TLG

Univariate analysis included 18 studies (n = 1,519) (20, 22, 24, 25, 27, 29, 31, 32, 34–40, 50–52) for MTV and 11 (n = 857) (22, 24, 25, 27, 29, 32, 37–40, 50) for TLG (**Figure 4**). High MTV (HR 1.80, 95% CI 1.40–2.33; GRADE: low) and TLG (HR 1.92, 95% CI 1.18–3.12; GRADE: moderate) both were significantly associated with inferior OS (**Supplementary Figures 7A**, **8A**). Egger’s test suggested potential publication bias (MTV, P < 0.01; TLG, P = 0.01), whereas Begg’s test showed no significant bias (**Supplementary Figures 7D**, **8D**). Leave-one-out sensitivity analysis indicated that the pooled estimates remained statistically significant (**Supplementary Figures 7C**, **8C**).

Multivariable analysis evaluated 11 studies (n = 743) (28, 29, 34, 36, 39, 42–46, 51) for MTV and 6 (n = 363) (29, 42, 44–48) for TLG (**Figure 4**). MTV remained significantly associated with OS (HR 1.14, 95% CI 1.03–1.26; GRADE: low), while TLG lost its statistical significance (HR 1.00, 95% CI 0.99–1.01; GRADE: moderate) (**Supplementary Figures 7B**, **8B**). Compared with univariate analysis, inter-study heterogeneity for both parameters decreased. Begg’s and Egger’s tests indicated that MTV remained consistent with univariate results (**Supplementary Figures 7E, F**).

### 3.5 Subgroup

**Figure 5:**
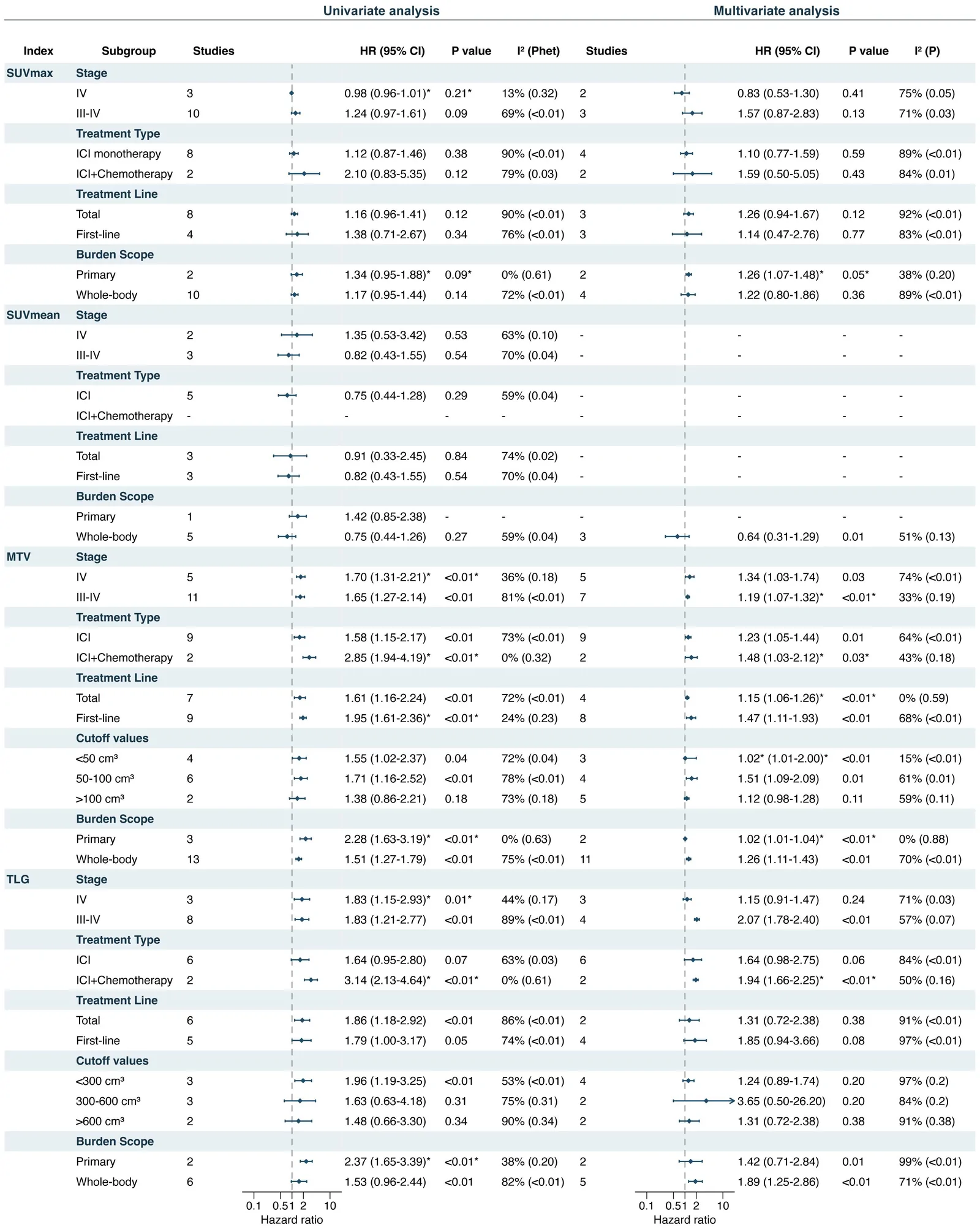
Univariate and multivariate meta-analysis of the impact of different clinical and imaging biomarkers on progression-free survival (PFS).

**Figure 6:**
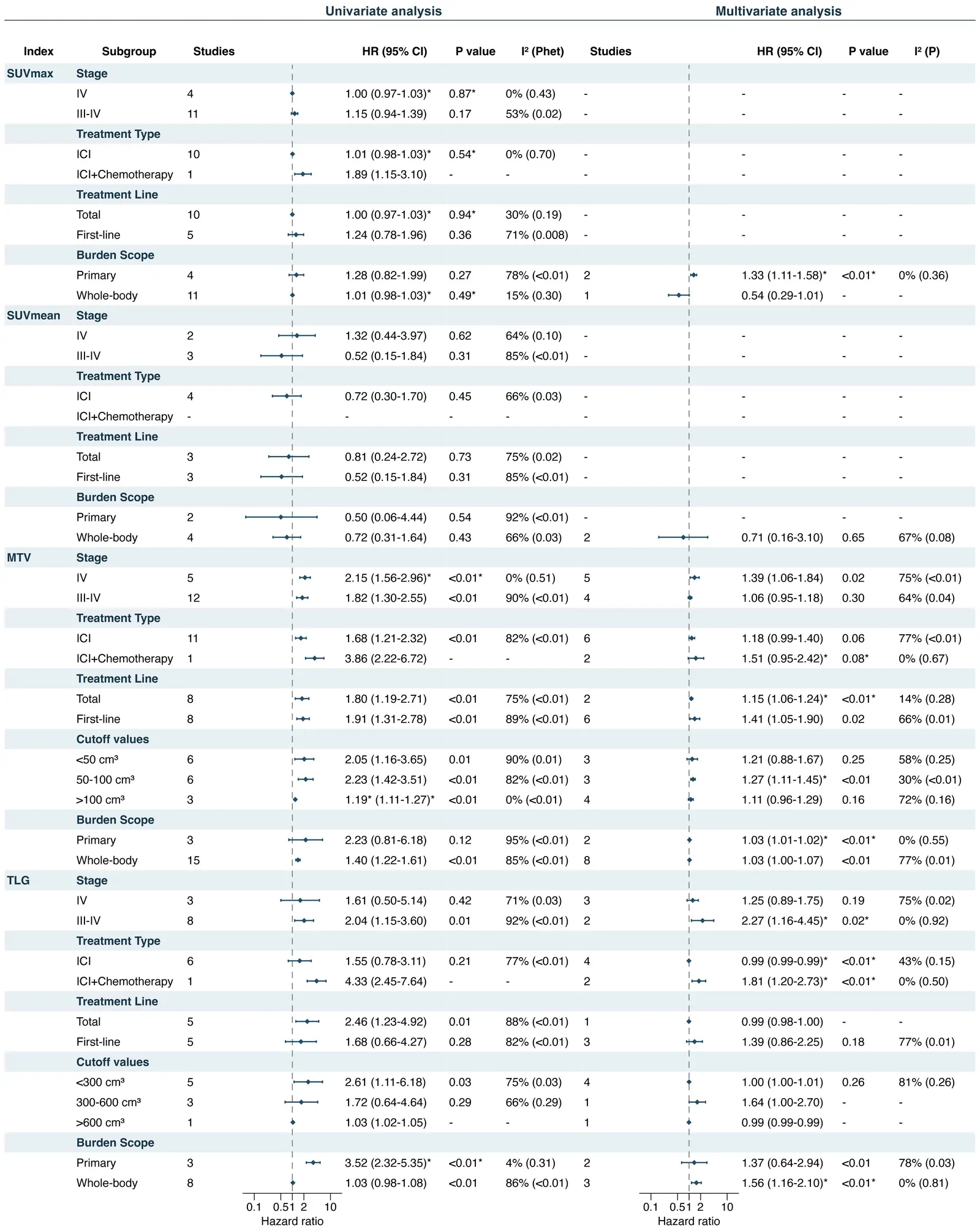
Univariate and multivariate meta-analysis of the impact of different clinical and imaging biomarkers on overall survival (OS).

Systematic subgroup analyses were performed to explore sources of heterogeneity and identify potential effect modifiers. Stratification was based on tumour stage, treatment type, treatment line, cut-off values, and tumor burden scope. Across most predefined clinical subgroups, both MTV and TLG maintained significant associations with prognostic outcomes (**Figures 5**, **6**).

Univariate subgroup analyses showed higher HRs for MTV and TLG in the chemoimmunotherapy group than in the ICI-monotherapy group. Among patients with stage IV NSCLC, high MTV was associated with shorter OS (HR = 2.15, 95% CI: 1.56–2.96), representing the largest effect across disease stages. The HR for TLG was also higher in later-line than first-line therapy (2.46). By measurement scope, primary-tumor MTV and TLG were significantly associated with OS, with pooled HRs of 2.28 and 3.52 (95% CI: 2.32–5.35), respectively. All MTV cutoff subgroups and the TLG cutoff subgroup below 300 cm³ were associated with PFS and OS in univariate analyses; however, after multivariate adjustment, only MTV within the 50–100 cm³ cutoff subgroup remained significant for both outcomes (*P* < 0.05). Adjustment reduced heterogeneity in several subgroups, particularly those receiving ICI monotherapy and those with stage III–IV disease. Primary-tumor MTV remained independently associated with PFS and OS without interstudy heterogeneity (both *I*² = 0%), whereas primary-tumor TLG lost significance. Conversely, whole-body TLG remained associated with PFS (HR = 1.89) and OS (HR = 1.56; *I*² = 0%).

## 4 Discussion

This study systematically evaluated the prognostic value of baseline [^18^F] FDG PET/CT metabolic parameters in patients with NSCLC receiving immunotherapy using univariate, multivariate, and multidimensional subgroup analyses. Pooled results revealed distinct predictive performance across parameters, SUVmax and SUVmean lacked clear prognostic value, whereas MTV and TLG, which reflect total metabolic tumor burden, showed stronger predictive ability. Multivariate analysis further identified high MTV as an independent predictor of shorter PFS and OS.

[^18^F] FDG uptake reflects both tumor metabolism and the immune microenvironment. Elevated SUV may represent the c-Myc–driven Warburg effect and metabolic activation of effector T cells (53); however, the limited spatial resolution of clinical PET/CT prevents reliable discrimination between tumor and immune signals (20). SUV is therefore not incorporated into the NCCN or ESMO prognostic frameworks (54, 55), and its predictive value appears limited to extreme cutoffs, without independent significance across conventional ranges (56). Consistently, baseline SUV was not associated with OS or PFS in this study. Previous meta-analyses combined univariate and multivariate estimates, potentially introducing statistical bias (57), whereas our findings remained robust after accounting for this confounding. Similarly, the univariate prognostic significance of SUVmax reported by Luo et al. (32) disappeared after adjustment for whole-body MTV (MTVwb), suggesting confounding by tumor burden and invasiveness.

Unlike SUV, which reflects local metabolic intensity, MTV and TLG quantify total metabolic tumor burden (TMTB). Subgroup analyses further indicated that anatomical measurement scope influenced their prognostic stability. In multivariate analyses of overall survival, heterogeneity in the MTV subgroup decreased from 95% for primary-tumor assessment to 77% for whole-body assessment. More strikingly, TLG lacked independent prognostic value when restricted to the primary tumor but regained predictive significance with no heterogeneity under whole-body assessment. These findings suggest that primary-tumor-only measurements may introduce model bias and statistical noise, supporting the preferential use of whole-body metabolic tumor burden in future prognostic evaluations of immunotherapy.

Beyond statistical stability, the clinical value of whole-body volumetric assessment may be biologically linked to the tumor microenvironment. Hypoxia-inducible factor 1α (HIF-1α) suppresses CD8+ T-cell activity through the CD73–adenosine axis, while the monocarboxylate transporter 4 (MCT4)–lactate pathway acidifies the microenvironment and promotes the recruitment of myeloid-derived suppressor cells (MDSCs) and regulatory T cells (Tregs) (58, 59). Thus, elevated MTV and TLG may indicate a hypoxic, immunosuppressive tumor milieu.In contrast to a previous meta-analysis (57), TLG showed a larger effect size than MTV and superior predictive performance in the cutoff subgroup below 300 cm³. However, its prognostic stability declined after multivariate adjustment. Because TLG integrates metabolic volume and intensity, it may enhance risk discrimination while amplifying heterogeneity arising from variations in SUV measurement protocols (60), thereby limiting its reliability as an independent prognostic factor. Effect estimates may also be inflated in small studies with inadequate confounder adjustment, as illustrated by Chardin et al. (50) (OS: TLG, HR = 5.37; MTV, HR = 5.05). Neither MTV nor TLG is currently included in routine NCCN or ESMO assessments, partly because standardized cutoff values remain unavailable (54, 55). Nevertheless, meta-analytic evidence suggests that a baseline MTV cutoff of 50–100 cm³ provides optimal risk stratification (61), consistent with the robust prognostic performance of this subgroup in our multivariate analysis. Overall, TLG may offer greater sensitivity for identifying aggressive phenotypes, whereas MTV provides a simpler and more stable volumetric measure. MTV may therefore be prioritized for clinical stratification, with TLG serving as a complementary marker.

In the immunochemotherapy subgroup, MTV and TLG showed greater prognostic value and lower heterogeneity than in the ICI-monotherapy subgroup. This advantage may partly reflect platinum-induced immunogenic cell death (ICD), characterized by calreticulin (CRT) exposure and high-mobility group box 1 (HMGB1) release (62, 63). Platinum-induced ICD may mitigate hypoxia-associated immune evasion by attenuating CD73–adenosine-mediated CD8+ T-cell suppression and promoting functional tumor-infiltrating lymphocytes, thereby disrupting immunosuppressive conditions maintained by MDSCs and Tregs. Because high MTV is associated with a profoundly immunosuppressive microenvironment (58, 59), immunochemotherapy and closer surveillance may warrant consideration in patients with a high baseline volumetric burden. MTV also showed stronger prognostic performance in patients with stage IV disease than in mixed-stage cohorts. Moreover, the prognostic predominance of TLG in later-line treatment was consistent with the findings of Dall’Olio et al. (64). Thus, a high baseline TLG may identify patients unlikely to benefit from later-line ICI monotherapy and support earlier consideration of combination regimens or treatment modification, while balancing therapeutic benefit against toxicity and resource use.

## 5 Limitation

This study has several limitations. First, significant inter-study heterogeneity remains. Although focusing on whole-body metabolic burden partially mitigated this, inconsistent image segmentation protocols and cross-center variations in scanner technology, reconstruction algorithms, and matrix sizes persist. These methodological differences systematically shift baseline MTV and TLG thresholds. Second, the absence of direct correlation data between metabolic parameters and key clinicopathological features in the primary studies precluded deeper subgroup or meta-regression analyses. Third, Egger’s test indicated potential publication bias toward positive outcomes; however, sensitivity analyses confirmed the overall robustness of our pooled results. Finally, because the included cohorts predominantly comprised patients with advanced (Stage III–IV) NSCLC, the generalizability of these findings to early-stage populations is limited, underscoring the need for large-scale prospective validation.

## 6 Conclusion

In NSCLC patients treated with ICIs, MTV serves as a robust prognostic biomarker, showing optimal performance within the 50-100cm^3^ range. Despite the instability of TLG following multivariate adjustment, it maintains efficacy in cohorts characterized by high heterogeneity and heavy pretreatment. Clinical translation of these metric hinges on standardized segmentation and validation in prospective multicenter trials.

## Data Availability

The original contributions presented in the study are included in the article/**Supplementary Material**. Further inquiries can be directed to the corresponding author.
