# Supplementary material for: Prognostic superiority of volumetric PET parameters (MTV and TLG) over SUV in advanced non-small-cell lung cancer: a systematic review and meta-analysis of univariate and multivariate evidence
Source: Front Immunol. 2026 Aug 25;17:1896967. doi: 10.3389/fimmu.2026.1896967 (PMC13550222; doi:10.3389/fimmu.2026.1896967)
Supplement: Supplementary file 1 [file Table1.docx]

**Prognostic Superiority of Volumetric PET Parameters (MTV and TLG) over SUV in Advanced Non-Small-Cell Lung Cancer: A Systematic Review and Meta-Analysis of Univariate and Multivariate Evidence**

**Supplementary Figure Legend:**

**Supplementary Figure 1:** Forest plot of a meta-analysis of the prognostic role of SUVmax. A: Univariate analysis for PFS; B: Multivariate analysis for PFS. Sensitivity analysis of a meta-analysis of the prognostic role of SUVmax. C: Univariate analysis for PFS; D: funnel plot of Univariate analysis.

**Supplementary Figure 2:** Forest plot of a meta-analysis of the prognostic role of SUVmean. A: Univariate analysis for PFS; B: Multivariate analysis for PFS.

**Supplementary Figure 3:** Forest plot of a meta-analysis of the prognostic role of MTV. A: Univariate analysis for PFS; B: Multivariate analysis for PFS. Sensitivity analysis of a meta-analysis of the prognostic role of MTV. C: Univariate analysis for PFS; E: Multivariate analysis for PFS; D: funnel plot of Univariate analysis; F: funnel plot of Multivariate analysis.

**Supplementary Figure 4:** Forest plot of a meta-analysis of the prognostic role of TLG. A: Univariate analysis for PFS; B: Multivariate analysis for PFS. Sensitivity analysis of a meta-analysis of the prognostic role of TLG. C: Univariate analysis for PFS; D: funnel plot of Univariate analysis.

**Supplementary Figure 5:** Forest plot of a meta-analysis of the prognostic role of SUVmax. A: Univariate analysis for OS; B: Multivariate analysis for OS. Sensitivity analysis of a meta-analysis of the prognostic role of SUVmax. C: Univariate analysis for OS; D: funnel plot of Univariate analysis.

**Supplementary Figure 6:** Forest plot of a meta-analysis of the prognostic role of SUVmean. A: Univariate analysis for OS; B: Multivariate analysis for OS.

**Supplementary Figure 7:** Forest plot of a meta-analysis of the prognostic role of MTV. A: Univariate analysis for OS; B: Multivariate analysis for OS. Sensitivity analysis of a meta-analysis of the prognostic role of MTV. C: Univariate analysis for OS; F: Multivariate analysis for OS; D: funnel plot of Univariate analysis; F: funnel plot of Multivariate analysis.

**Supplementary Figure 8:** Forest plot of a meta-analysis of the prognostic role of TLG. A: Univariate analysis for OS; B: Multivariate analysis for OS. Sensitivity analysis of a meta-analysis of the prognostic role of TLG.C: Univariate analysis for OS; D: funnel plot of Univariate analysis.

**Supplementary Figure 1** Forest plot of a meta-analysis of the prognostic role of SUVmax. A: Univariate analysis for PFS; B: Multivariate analysis for PFS. Sensitivity analysis of a meta-analysis of the prognostic role of SUVmax. C: Univariate analysis for PFS; D: funnel plot of Univariate analysis.

| A |  | B |  |
| --- | --- | --- | --- |
| C |  | D |  |

**Supplementary Figure 2** Forest plot of a meta-analysis of the prognostic role of SUVmean. A: Univariate analysis for PFS; B: Multivariate analysis for PFS.

| A |  | B |  |
| --- | --- | --- | --- |

**Supplementary Figure 3** Forest plot of a meta-analysis of the prognostic role of MTV. A: Univariate analysis for PFS; B: Multivariate analysis for PFS. Sensitivity analysis of a meta-analysis of the prognostic role of MTV. C: Univariate analysis for PFS; E: Multivariate analysis for PFS; D: funnel plot of Univariate analysis; F: funnel plot of Multivariate analysis.

| A |  |  | B |  |
| --- | --- | --- | --- | --- |
| C |  |  | D |  |
| E |  |  | F |  |

**Supplementary Figure 4** Forest plot of a meta-analysis of the prognostic role of TLG. A: Univariate analysis for PFS; B: Multivariate analysis for PFS. Sensitivity analysis of a meta-analysis of the prognostic role of TLG. C: Univariate analysis for PFS; D: funnel plot of Univariate analysis.

| A |  | B |  |
| --- | --- | --- | --- |
| C |  | D |  |

**Supplementary Figure 5** Forest plot of a meta-analysis of the prognostic role of SUVmax. A: Univariate analysis for OS; B: Multivariate analysis for OS. Sensitivity analysis of a meta-analysis of the prognostic role of SUVmax. C: Univariate analysis for OS; D: funnel plot of Univariate analysis.

| A |  | B |  |
| --- | --- | --- | --- |
| C |  | D |  |

**Supplementary Figure 6** Forest plot of a meta-analysis of the prognostic role of SUVmean. A: Univariate analysis for OS; B: Multivariate analysis for OS.

| A |  | B |  |
| --- | --- | --- | --- |

**Supplementary Figure 7** Forest plot of a meta-analysis of the prognostic role of MTV. A: Univariate analysis for OS; B: Multivariate analysis for OS.Sensitivity analysis of a meta-analysis of the prognostic role of MTV. C: Univariate analysis for OS; F: Multivariate analysis for OS; D: funnel plot of Univariate analysis; F: funnel plot of Multivariate analysis.

| A |  | B |  |
| --- | --- | --- | --- |
| C |  | D |  |
| E |  | F |  |

**Supplementary Figure 8** Forest plot of a meta-analysis of the prognostic role of TLG. A: Univariate analysis for OS; B: Multivariate analysis for OS. Sensitivity analysis of a meta-analysis of the prognostic role of TLG.C: Univariate analysis for OS; D: funnel plot of Univariate analysis.

| A |  | B |  |
| --- | --- | --- | --- |
| C |  | D |  |

**Supplementary Tables:**

**Supplementary Table 1**: Search strategy (PubMed as an example).

**Supplementary Table 2**: GRADE evidence quality assessment and prognostic impact of PET/CT metabolic parameters on PFS in NSCLC patients.

**Supplementary Table 3**: GRADE evidence quality assessment and prognostic impact of PET/CT metabolic parameters on OS in NSCLC patients.

**Supplementary Table 1** Search strategy (PubMed as an example).

| **Search Step** | **Search Element** | **Search Items (PubMed format)** |
| --- | --- | --- |
| Step 1 | Imaging Technology: PET/CT | "18F-FDG"[Title/Abstract] |
|  |  | "fluorodeoxyglucose"[Title/Abstract] |
|  |  | "positron emission tomography"[Title/Abstract] |
| Step 2 | Population/Disease: NSCLC | "NSCLC" |
|  |  | "non-small-cell lung cancer"[Title/Abstract] |
| Step 3 | Intervention/Therapy: ICIs | "immune checkpoint inhibitor"[Title/Abstract] |
|  |  | "immunotherapy"[Title/Abstract] |
|  |  | "Nivolumab" |
|  |  | "pembrolizumab" |
|  |  | "atezolizumab" |
|  |  | "ICIs"[Title/Abstract] |
| Step 4 | Final Union | #1 AND #2 AND #3 |

**Supplementary Table 2 GRADE evidence quality assessment and prognostic impact of PET/CT metabolic parameters on PFS in NSCLC patients.**

| Index | Analysis | Number of Studies | Certainty assessment | | | | | | Effect | Certainty | Importance |
| --- | --- | --- | --- | --- | --- | --- | --- | --- | --- | --- | --- |
|  |  |  | Study design | Risk of bias | Inconsistency | Indirectness | Imprecision | Other considerations | Hazard Ratio (95%CI) |  |  |
| SUVmax | Univariate | 13 | non-randomised | serious | serious | not serious | serious | none | 1.2(0.98-1.46) | ⨁◯◯◯ Very Low | IMPORTANT |
|  | Multivariate | 6 | non-randomised | not serious | serious | not serious | serious | none | 1.19(0.84-1.68) | ⨁⨁◯◯ Low | IMPORTANT |
| SUVmean | Univariate | 6 | non-randomised | serious | serious | not serious | serious | none | 0.85(0.52-1.39) | ⨁◯◯◯ Very Low | IMPORTANT |
|  | Multivariate | 3 | non-randomised | not serious | serious | not serious | serious | none | 0.63(0.31-1.28) | ⨁⨁◯◯ Low | IMPORTANT |
| MTV | Univariate | 17 | non-randomised | serious | serious | not serious | not serious | publication bias strongly suspected strong association | 1.71(1.39-2.11) | ⨁⨁◯◯ Low | IMPORTANT |
|  | Multivariate | 14 | non-randomised | not serious | serious | not serious | not serious | publication bias strongly suspected | 1.21(1.07-1.36) | ⨁⨁◯◯ Low | IMPORTANT |
| TLG | Univariate | 11 | non-randomised | serious | serious | not serious | not serious | strong association | 1.84(1.31-2.57) | ⨁⨁⨁◯ Moderate | IMPORTANT |
|  | Multivariate | 9 | non-randomised | not serious | serious | not serious | not serious | none | 1.46(1.10-1.94) | ⨁⨁⨁◯ Moderate | IMPORTANT |

**Supplementary Table 3** GRADE evidence quality assessment and prognostic impact of PET/CT metabolic parameters on OS in NSCLC patients.

| Index | Analysis | Number of Studies | Certainty assessment | | | | | | Effect | Certainty | Importance |
| --- | --- | --- | --- | --- | --- | --- | --- | --- | --- | --- | --- |
|  |  |  | Study design | Risk of bias | Inconsistency | Indirectness | Imprecision | Other considerations | Hazard Ratio (95%CI) |  |  |
| SUVmax | Univariate | 15 | non-randomised | serious | not serious | not serious | serious | none | 1.01(0.98-1.03) * | ⨁⨁◯◯ Low | CRITICAL |
|  | Multivariate | 3 | non-randomised | not serious | serious | not serious | serious | none | 0.97(0.57-1.64) | ⨁⨁◯◯ Low | CRITICAL |
| SUVmean | Univariate | 6 | non-randomised | serious | serious | not serious | serious | none | 0.65(0.29-1.44) | ⨁◯◯◯ Very Low | CRITICAL |
|  | Multivariate | 2 | non-randomised | not serious | serious | not serious | serious | none | 0.71(0.16-3.10) | ⨁⨁◯◯ Low | CRITICAL |
| MTV | Univariate | 18 | non-randomised | serious | serious | not serious | not serious | publication bias strongly suspected strong association | 1.80(1.40-2.33) | ⨁⨁◯◯ Low | CRITICAL |
|  | Multivariate | 11 | non-randomised | not serious | serious | not serious | not serious | publication bias strongly suspected | 1.14(1.03-1.26) | ⨁⨁◯◯ Low | CRITICAL |
| TLG | Univariate | 11 | non-randomised | serious | serious | not serious | not serious | strong association | 1.92(1.18-3.12) | ⨁⨁⨁◯ Moderate | CRITICAL |
|  | Multivariate | 7 | non-randomised | not serious | serious | not serious | not serious | none | 1.00(0.99-1.01) | ⨁⨁⨁◯ Moderate | CRITICAL |
